# Transcriptomic analysis to reveal the differentially expressed miRNA targets and their miRNAs in response to *Ralstonia solanacearum* in ginger species

**DOI:** 10.1186/s12870-021-03108-0

**Published:** 2021-07-29

**Authors:** Mohandas Snigdha, Duraisamy Prasath

**Affiliations:** ICAR-Indian Institute of Spices Research, Kozhikode, Kerala 673012 India

**Keywords:** Bacterial wilt, Ginger, miRNA, Mango ginger, Ralstonia, Target genes

## Abstract

**Background:**

Bacterial wilt is the most devastating disease in ginger caused by *Ralstonia solanacearum*. Even though ginger (*Zingiber officinale*) and mango ginger (*Curcuma amada*) are from the same family Z*ingiberaceae*, the latter is resistant to *R. solanacearum* infection. MicroRNAs have been identified in many crops which regulates plant-pathogen interaction, either through silencing genes or by blocking mRNA translation. However, miRNA’s vital role and its targets in mango ginger in protecting bacterial wilt is not yet studied extensively. In the present study, using the “psRNATarget” server, we analyzed available ginger (susceptible) and mango ginger (resistant) transcriptome to delineate and compare the microRNAs (miRNA) and their target genes (miRTGs).

**Results:**

A total of 4736 and 4485 differential expressed miRTGs (DEmiRTGs) were identified in ginger and mango ginger, respectively, in response to *R. solanacearum*. Functional annotation results showed that mango ginger had higher enrichment than ginger in top enriched GO terms. Among the DEmiRTGs, 2105 were common in ginger and mango ginger. However, 2337 miRTGs were expressed only in mango ginger which includes 62 defence related and upregulated miRTGs. We also identified 213 miRTGs upregulated in mango ginger but downregulated in ginger, out of which 23 DEmiRTGS were defence response related. We selected nine miRNA/miRTGs pairs from the data set of common miRTGs of ginger and mango ginger and validated using qPCR.

**Conclusions:**

Our data covered the expression information of 9221 miRTGs. We identified nine miRNA/miRTGs key candidate pairs in response to *R. solanacearum* infection in ginger. This is the first report of the integrated analysis of miRTGs and miRNAs in response to *R. solanacearum* infection among ginger species. This study is expected to deliver several insights in understanding the miRNA regulatory network in ginger and mango ginger response to bacterial wilt.

**Supplementary Information:**

The online version contains supplementary material available at 10.1186/s12870-021-03108-0.

## Background

India is rich in spices and has been regarded as the “Spice Bowl of the World”. Ginger (*Zingiber officinale* Rosc.), the major spice and medicinal crop, is cultivated mainly in tropical and subtropical regions of India. The rhizomes contain several bioactive compounds used as a flavouring agent, herbal medicine and is also employed in the perfume industry [1, 2]. India is the major producer of ginger, accounting for 31% of the world production [3]. Several reports have been published regarding the crop loss due to bacterial wilt caused by *Ralstonia solanacearum* [2]. Bacterial wilt is considered as a major disease especially in regions with warm climates [4]. India has crossed yield losses of more than 50% due to this infection [5]. Around the world, the *R. solanacearum*, a soil-borne bacterium, causes bacterial wilt in many plant families such as tomato, potato, pepper, peanut, banana and eggplant [6]. This gram-negative bacterium infects plants through axils of secondary roots, which later invades cortex and then colonizes in the xylem vessels which causes wilt symptoms and death [7].

Many recent studies have increasingly concentrated on host plant resistance than traditional chemical treatments, which seems to be more powerful and economical in controlling bacterial wilt infection [8]. Several enhanced resistance varieties of potato, tomato, peanut, eggplant, and banana have been successfully generated by transforming the resistant gene to the plant [7, 9]. Research has been carried out to identify the resistance source to Ralstonia induced bacterial wilt in ginger. In a study, the ICAR-Indian Institute of Spices Research, Kozhikode, Kerala, reported mango ginger (*Curcuma amada* Roxb.) from the ginger family *Zingiberaceae*, with a high level of resistance against bacterial wilt. Comparative transcriptomics of ginger and mango ginger during bacterial wilt has resulted in identifying a considerable number of candidate genes [10].

Earlier reports states that microRNAs (miRNAs) are hypersensitive to diverse physiological processes such as abiotic or biotic stress [11, 12]. miRNAs which are approximately 21-nucleotide noncoding RNAs, serve a crucial role in post-transcriptional gene regulation by degrading target mRNAs in plants. Previous researchers have identified several plant miRNAs and their targets, which are related to biotic stress. This supports that miRNAs are crucial to the stress response of plants [13]. Thus far, the miRNA expression profiles of ginger under bacterial stress conditions have rarely been reported. Efforts to identify bacterial wilt responsive miRNAs and determine their expression patterns would improve our understanding of their stress adaptation functions.

Several researchers reported that pathogen attack in plants induce numerous miRNAs and later control and contribute towards the reprogramming of gene expression [12–19]. It was reported that during wheat-stripe rust infection, several variants of known miRNAs and 163 candidate novel wheat miRNAs were differentially expressed [12]. Much research has been carried out to overexpress significant miRNAs to enhance disease resistance in susceptible plants. Overexpressing miR319b in rice found to be a positive regulator of the rice defence response against the blast disease [14]. Differential expression of miRNAs was observed in wheat and barley after infection with powdery mildew [15, 16]. The introduction of artificial miRNA has been carried out in several plants against viral infection, such as wheat, maize, tomato, and grape wine [17–19].

This study employed already available transcriptome to investigate and elucidate a detailed and in-depth characterization of miRTGs and their expression in ginger (susceptible) and mango ginger (resistant) transcriptome during *R. solanacearum* infection. We identified miRTGs and their known miRNAs involved in defence-related, plant-pathogen interaction and disease resistance. Our results provided valuable information to reveal the molecular mechanism of miRNAs and their targets in resisting mango ginger against *R. solanacearum* infection.

## Results

### Identification of miRTGs

**Fig. 1:**
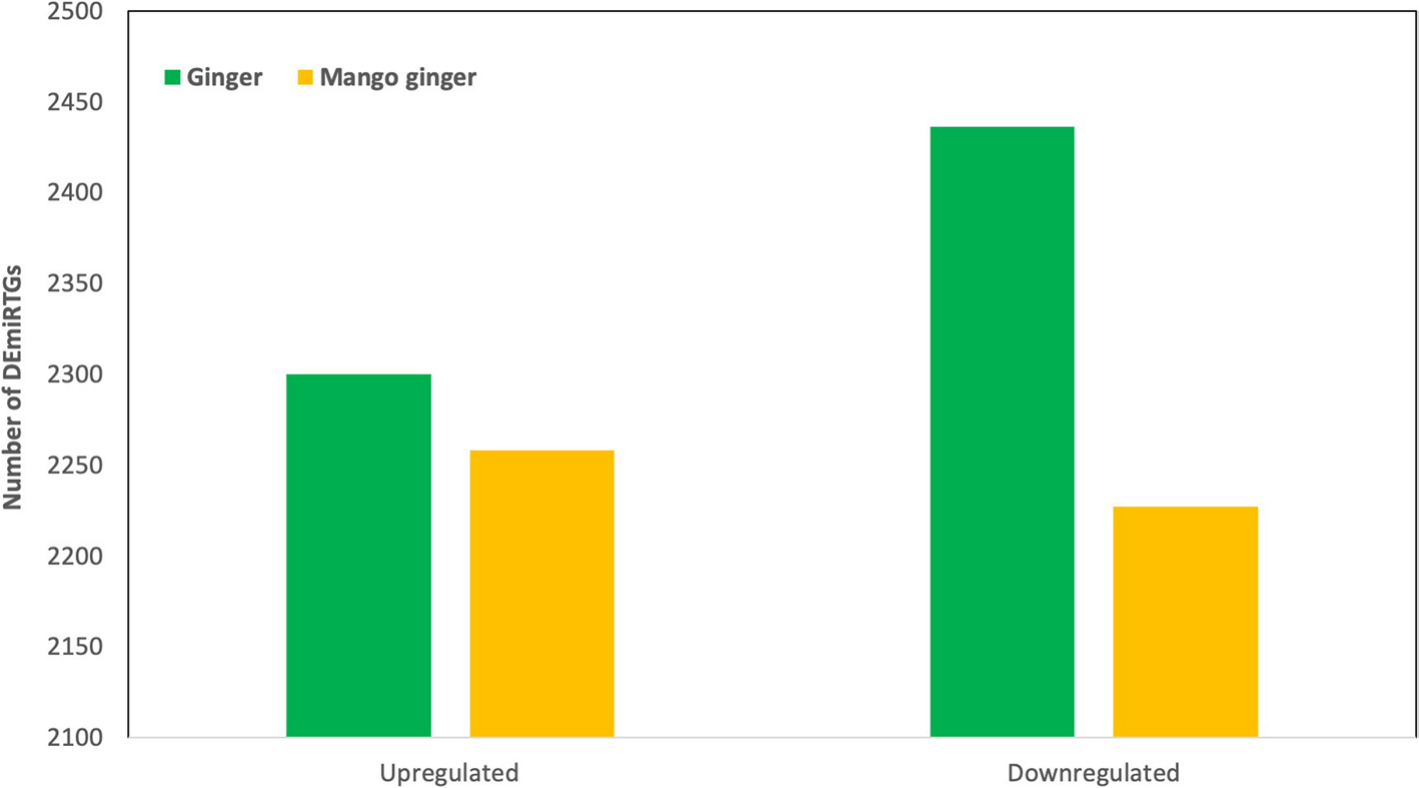
Numbers of differentially expressed total miRTGs in response to bacterial wilt in ginger and mango ginger

Already available ginger and mango ginger RNA-Seq data from our lab was utilized to reconstruct transcriptome de novo. A total of 80,496,326 and 66,561,960 raw reads, accounting approximately for mango ginger and ginger, respectively, were generated (Table 1). The mango ginger assembly was represented by 307,952 contigs with an average of 845.32 bps, while the ginger assembly was represented by 303,878 contigs with an average contig size of 692.32 bps. The N50 for mango ginger and ginger with de novo assemblies were 1256 and 1005, respectively. These assembled contigs were further used to identify known miRTGs. After carefully considering the alignment results, we located a total of 4736 and 4485 DEmiRTGs (significant *p*-value <  = 0.05 and having functional annotation) in ginger and mango ginger, respectively (Fig. 1).

**Table 1:** Assembly summary of transcripts of mango ginger and ginger

	Mango ginger	Ginger
Number of reads	80,496,326	66,561,960
Average read length	100	100
Average GC per reads	44	48
Number of contigs	307,952	303,878
Mean contig length	845.32	692.32
Maximum contig length	15,525	23,113
N50	1,256	1,005
N100	201	201

### Identification of miRNAs from miRTGs

**Fig. 2:**
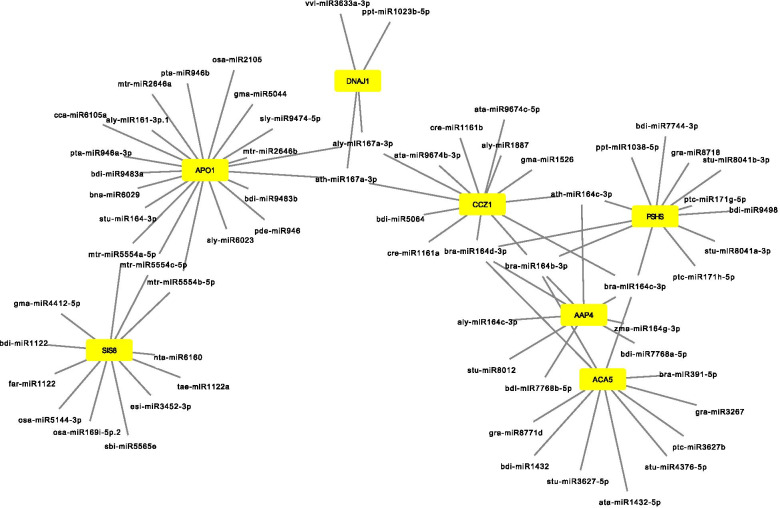
Regulation network constructed with selected miRTGs and corresponding miRNAs. Yellow indicates miRTGs and nodes indicates corresponding miRNAs

The ‘psRNATarget’ server was utilized to map miRNAs from miRTGs. Each miRTG provided a varying number of miRNAs. In ginger, maximum miRNAs (1193) were identified for the target, miRTG UBC24_ARATH. Among ginger miRTGs, 271 did not show any corresponding miRNAs. In mango ginger, miRTG NFYA7_ARATH had the maximum number of miRNAs (320). No miRNAs were identified for 315 mango ginger miRTGs. Each miRTGs had targets for several miRNAs and vice versa and they combined to form a complex network (Fig. 2; Supplementary data 1).

### Differential expression of miRTGs

**Fig. 3:**
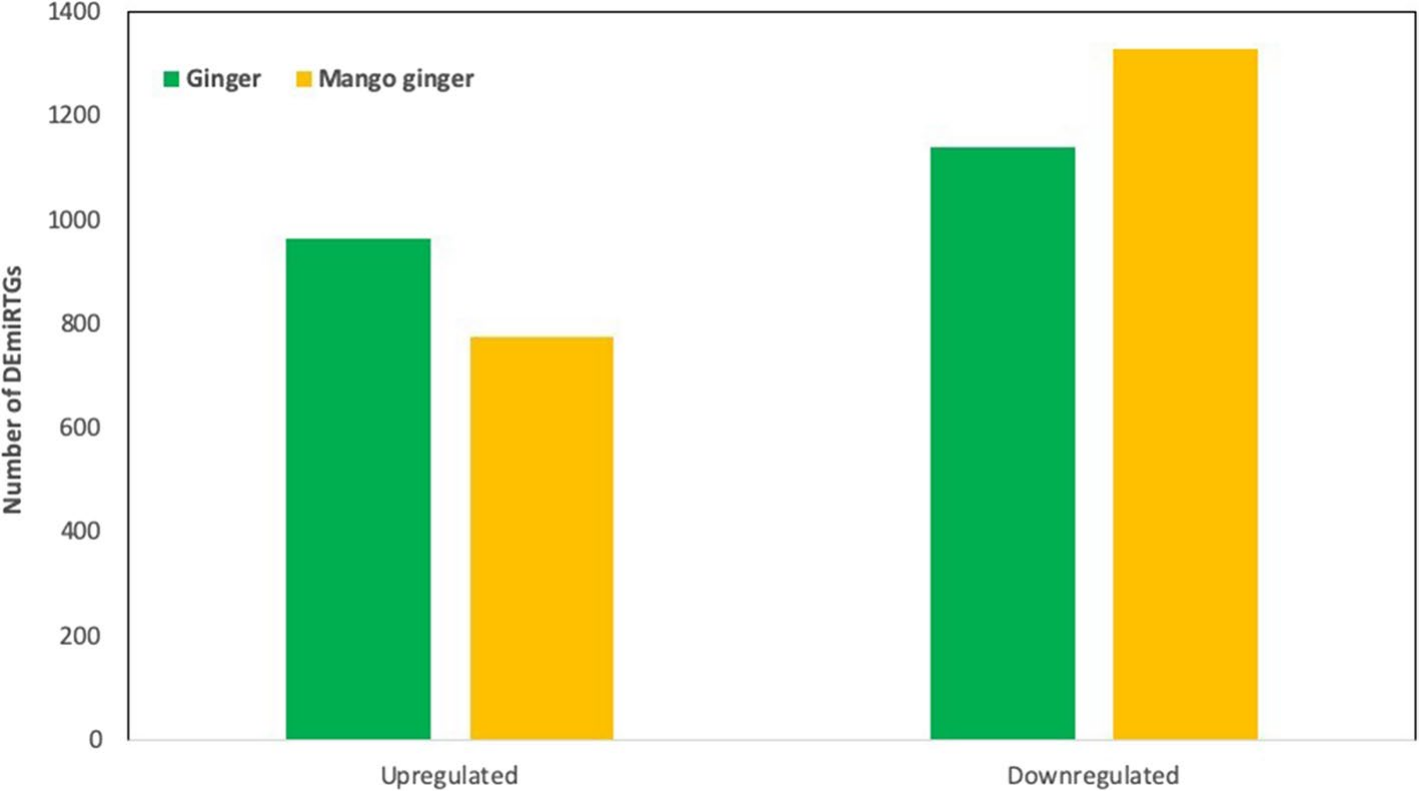
Numbers of differentially expressed common miRTGs in response to bacterial wilt in ginger and mango ginger

**Fig. 4:**
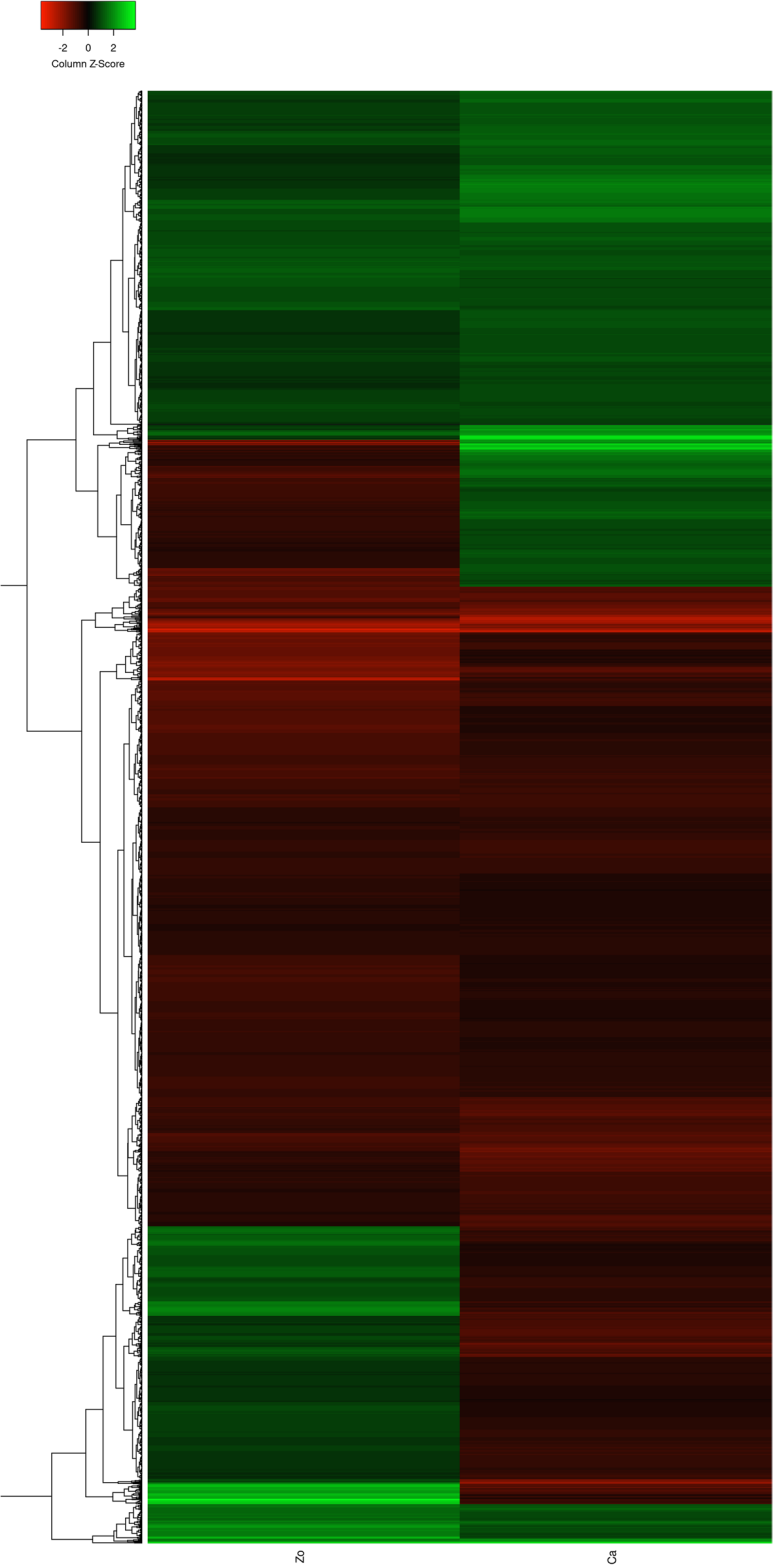
Heatmap showing the expression levels of the common DEmiRTGs of Zo (ginger) versus Ca (mango ginger). The color code indicates relative abundance, ranging from red (low abundance) to green (high abundance)

We identified 4736 differentially expressed miRTGs in ginger (susceptible), out of which 2300 were upregulated and 2436 were downregulated. In mango ginger (resistant), out of 4485 DEmiRTGs, 2258 were upregulated, and 2227 were downregulated (Fig. 1). Among the total 9921 DEmiRTGs in response to *R. solanacearum*, 2105 were common in ginger and mango ginger. The common DEmiRTGs revealed, 965 upregulated and 1140 downregulated in ginger and 776 upregulated, and 1329 downregulated in mango ginger (Fig. 3). When compared with ginger, 2337 miRTGs were expressed only in mango ginger. Moreover, we also observed 215 miRTGs upregulated in resistant mango ginger but downregulated in susceptible ginger (Fig. 4). Nine miRTGs were selected based on their function and fold change from the common miRTGs differentially expressed in both plants. Primers were designed for each target genes and its miRNAs (Tables 2 and 3). The real-time PCR analysis of these miRTGs genes and their miRNAs revealed their differential expression among the two ginger species.

**Table 2:** Primer designed for target genes to perform qPCR experiments

miRTGs	Primer sequence
zo-PRT6 forward	CTTCGGCGTGCTTATTGGAA
zo-PRT6 reverse	CCATCGAGGTAGCTGGACAT
zo-FRS6 forward	CCAGAGCCCAAGTATCGGAA
zo-FRS6 reverse	CAGGGTGGTTGTGGTCAATG
zo-RPS2 forward	TTGCTCGCTTCACTGAGTTG
zo-RPS2 reverse	GCCGCTTCAATCCTTCCAAT
zo-WRKY19 forward	ACCAAGAGTGCAGAAGGTCA
zo-WRKY19 reverse	ATGTGCCTTGCAGAAATGGG
zo-ABCC5 forward	CCAGCTAAGAGACGCTGAGA
zo-ABCC5 reverse	ACGCAGATACCAAAGGCAAC
zo-4CCL1 forward	CTTCTCTCCTCGCGTCTGAT
zo-4CLL1 reverse	AGCCGGAACTAGCTATCGTC
zo-DNAJ1 reverse	ATGCCCTGAAGGAGGGAATG
zo-DNAJ1 forward	ACTTCCTCCACCTCCAAAGG
zo-AAP4 forward	TTGTCATCCACCTTGTCGGA
zo-AAP4 reverse	GGGTGATGAACTCGCTCTTG
zo-ABCG11 forward	GGAACGGTGTTGGATTCAGG
zo-ABCG11 reverse	TCAGCAGCCTTAGCCAGTAG
ca-PRT6 forward	ATGCATGCTTGGTGAGTCTG
ca-PRT6 reverse	GCCTGCCCATTGTTCTTAGG
ca-FRS6 forward	CCATCATGGCCAGTTGGTTT
ca-FRS6 reverse	TCAGTAATGAGGGCCTTGGG
ca-RPS2 forward	GGGAACCTAGCCCTCCAAAT
ca-RPS2 reverse	CGTATCCAGCTTCACGCAAT
ca-WRKY19 forward	AGGCCGGACTAGTTACTGTG
ca-WRKY19 reverse	CACAATGTTTGCCACCTCCA
ca-ABCC5 forward	GCTGCTCCTCTTCTTCCTGA
ca-ABCC5 reverse	CAAACCGACCGAAACCTGAA
ca-4CLL1 forward	CGTCAAGTTCAACGGACCTC
ca-4CLL1reverse	TAGCCGGAACTAGCAATCGT
ca-DNAJ1 forward	ACGAGATGGTGCTGACGTAT
ca-DNAJ1 reverse	AGTATTGTCCTGGCTGCACT
ca-AAP4 forward	ATCTCTGCGGACTCATCCAG
ca-AAP4 reverse	GGCCTTTCTCATGGAAGCAG
ca-ABCG11 forward	CTACTGGCTAAGGCTGCTGA
ca-ABCG11 reverse	AGACGAATGAAGCACATGCC
Actin forward	TAGGTGCCCAGAGGTTCTATT
Actin reverse	ACCGCTAAGCACCACATTAC

**Table 3:** Primers designed to perform qPCR experiments with the shortlisted nine miRNAs

miRNA	Stem loop primer	Forward primer
osa-miR169	GTTGGCTCTGGTGCAGGGTCCGAGGTATTCGCACCAGAGCCAAC GAATGG	GTGGGCATCATCCATCCTA
ppt-miR1223	GTTGGCTCTGGTGCAGGGTCCGAGGTATTCGCACCAGAGCCAAC TAGAGG	GGGGTTGTAGAGTCATGCA
aly-miR398	GTTGGCTCTGGTGCAGGGTCCGAGGTATTCGCACCAGAGCCAAC CATATG	GTTGGGGTCGACATGAGAA
gra-miR482	GTCGTATCCAGTGCAGGGTCCGAGGTATTCGCACTGGATACGACTTGGAA	AACACGCTTCCCAAAACCTC
gma-miR4415	GTTGGCTCTGGTGCAGGGTCCGAGGTATTCGCACCAGAGCCAAC CCATGT	GTTTGGGTTGATTCTCATCACA
mtr-miR5261	GTTGGCTCTGGTGCAGGGTCCGAGGTATTCGCACCAGAGCCAAC AGCCAA	GGGGTCATTGTAGATGGCT
ath-miR167	GTTGGCTCTGGTGCAGGGTCCGAGGTATTCGCACCAGAGCCAAC CCAGAT	GTTTGAAGCTGCCAGCATG
ath-miR1886	GTTGGCTCTGGTGCAGGGTCCGAGGTATTCGCACCAGAGCCAAC TTTTCT	GGGGGTGAGAAGAAGAAGA
mtr-miR164	GTTGGCTCTGGTGCAGGGTCCGAGGTATTCGCACCAGAGCCAAC TGCACG	GTTTTGGAGAAGCAGGGCA
Reverse common	GTCGTATCCAGTGCAGGGT	

### GO and KEGG pathways of DEmiRTGs

**Fig. 5:**
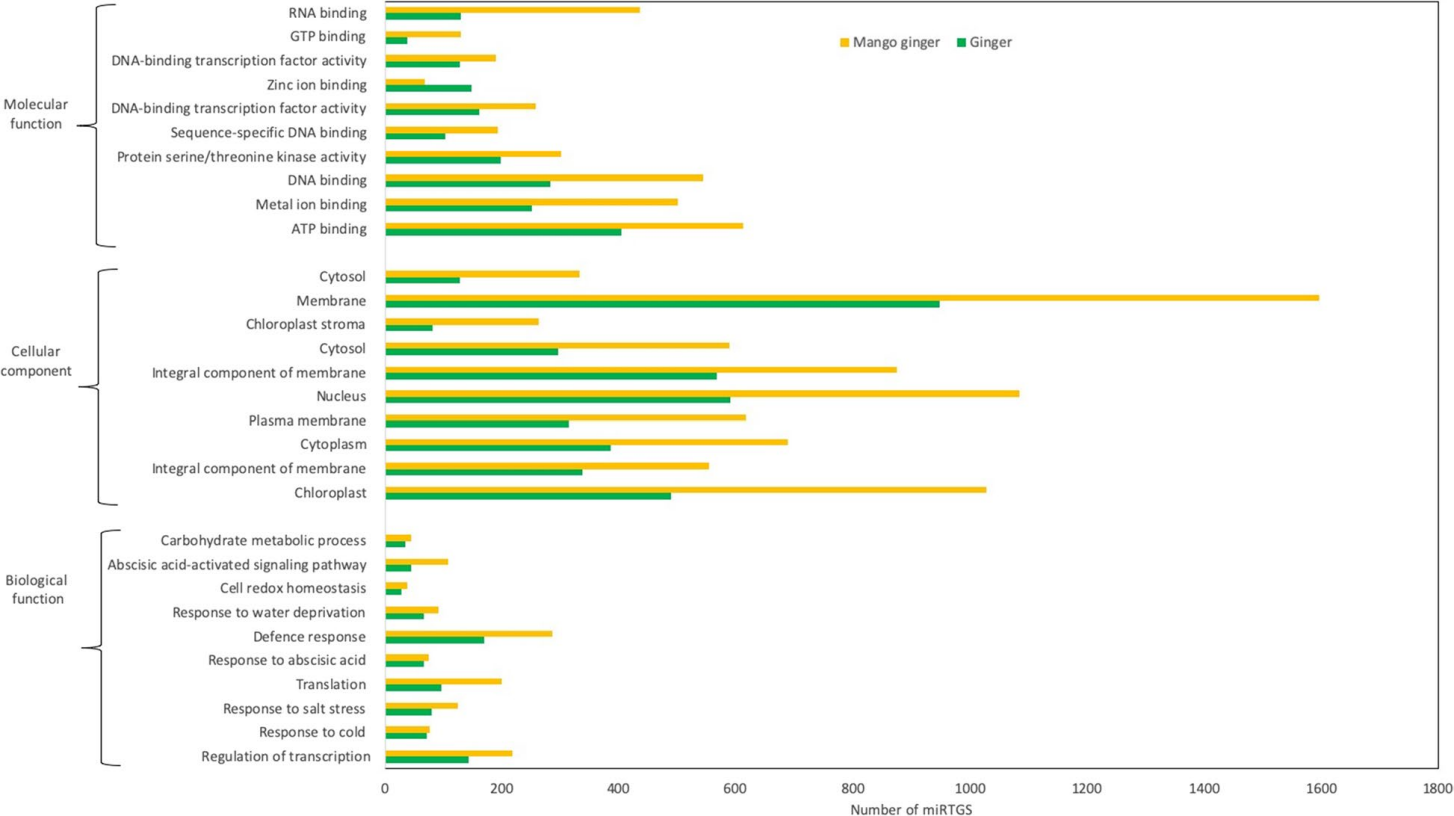
GO analysis of DEmiRTGs in response to bacterial wilt in ginger and mango ginger

Gene ontology (GO) analysis of DEmiRTGs was performed and grouped into three categories: biological process, cellular component, and molecular function. In the biological function category “defence response” represented the top term in ginger and mango ginger. The most abundant terms in cellular component category were “membrane” in ginger and mango ginger. In the molecular function, the GO term “ATP binding” was enriched in ginger and mango ginger (Fig. 5).

The GO analysis of upregulated DEmiRTGs expressed exclusively in mango ginger showed maximum enrichment in defence response (120), out of which 29 were related to “defence response in bacterium”. Among the 2105 common miRTGs, 45 and 71 were “defence response” GO terms upregulated in ginger and mango ginger, respectively. There were 213 miRTGs which were upregulated in mango ginger but downregulated in ginger. Among them 23 GO terms were related to “defense response”, which was the highest among all GO terms. Also, 10 GO terms were related to “defence response in bacterium” (Table 4).

**Table 4:** Differential expressed defense related miRTGs in response to *Ralstonia solanacearum*

Gene annotation	No. of genes	Ginger upregulated	Mango ginger upregulated	Target genes
Abscisic acid signalling	7	2	2	FERON_ARATH, UBA2A_ARATH, EDR1_ARATH, CDPKS_ARAT
Activation of protein kinase	4	2	4	M3K3A_ARATH
Autophagosome assembly	1	0	0	ATG7_ARATH
Brassinosteroid mediated signaling pathway	1	1	1	GLO1_ARATH
Defense response to bacterium	13	12	13	Y2766_ARATH, WRK53_ARATH, POP3_ARATH, NPR2_ORYSJ, SIR4_ARATH, P2C59_ARATH, RENT3_ARATH, BAHL2_ORYSI, CRK25_ARATH
Cellular response to jasmonic acid	7	3	7	HNRPQ_ARATH
Cellular response to osmotic stress	1	1	1	NTL9_ARATH
Cellular response to water deprivation	2	2	2	CP41B_ARATH
Ceramide biosynthetic process	2	2	2	IPCS_ORYSJ
Defense response	24	14	24	LRR2_ARATH, PBL17_ARATH, ERG1_ORYSJ, DRL17_ARATH, WRK19_ARATH, PBL19_ARATH, PBL3_ARATH, PBL16_ARATH, EDR2L_ARATH, LRKS5_ARATH, RPS2_ARATH, DGK5_ARATH, ERF82_ARATH, MKP1_ARATH, LRR1_ARATH
Defense response to fungus	7	4	7	CML36_ARATH, BBD1_ARATH, PRT6_ARATH, C3H47_ARATH
Carbohydrate metabolism process	2	2	2	E1314_ARATH
Ethylene signaling pathway	4	3	4	EDR2_ARATH

**Fig. 6:**
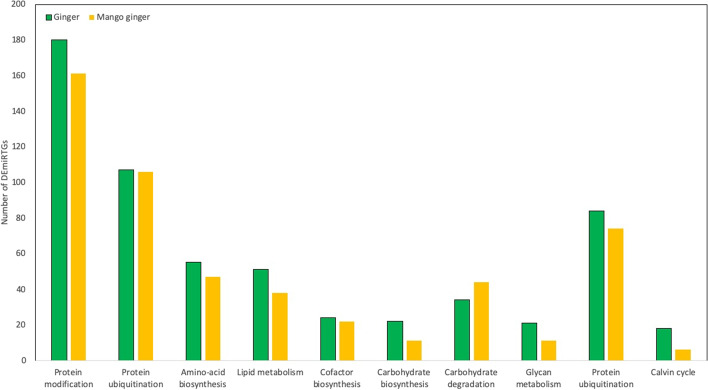
KEGG pathway analysis of DEmiRTGs in response to bacterial wilt in ginger and mango ginger

The top 10 enriched pathways regulated by Ralstoina are summarized in Fig. 6. The most enriched pathways were related to protein and amino acid pathways such as “protein modification” and “protein ubiquitination”. Interestingly, among the top 10 enriched pathways, we observed that the number of DEmiRTGs in mango ginger was more than ginger in the carbohydrate degradation and glycolysis pathways. Thus, indicating the difference and significance of carbohydrate metabolism in giving resistance against bacterial wilt.

### Validation and correlation analysis of miRTGs expression profiles and their miRNAs

To specify the roles of miRNAs in response to bacterial wilt, the expression profiles of nine miRNAs and miRTGs common in both ginger species were analysed.

**Fig. 7:**
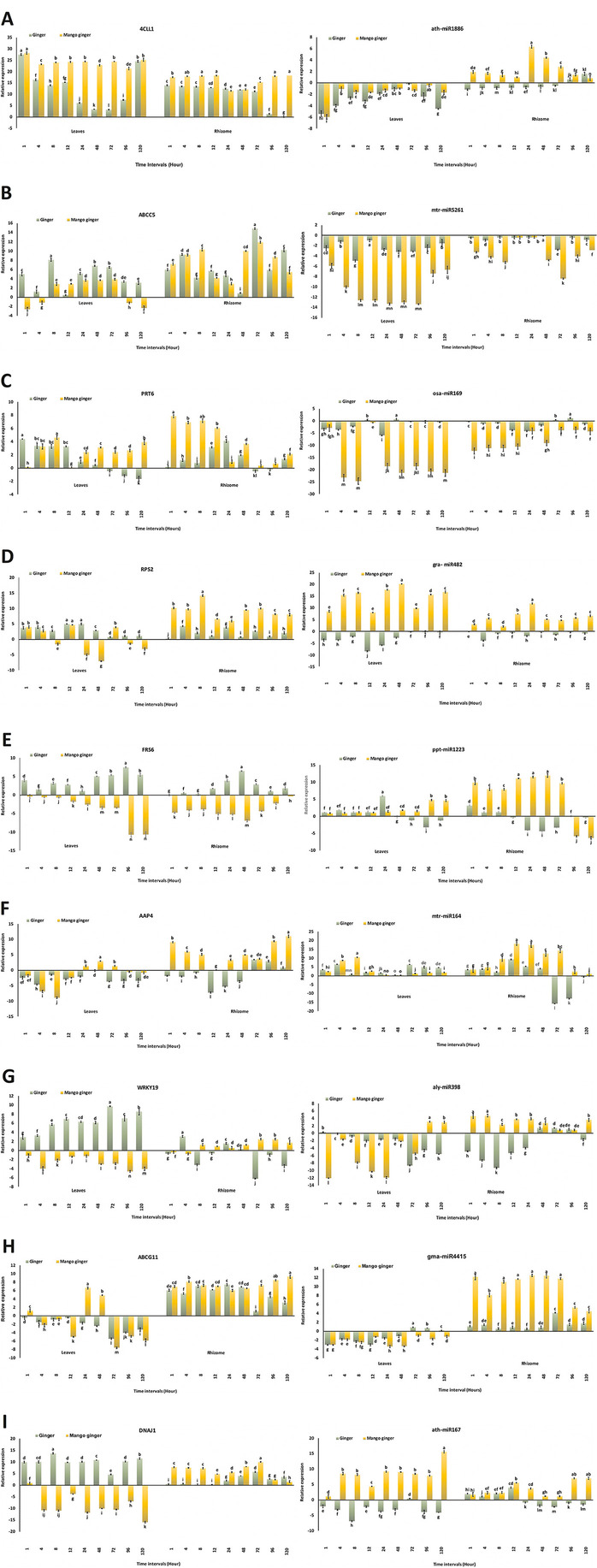
Normalized relative gene expression levels of nine miRNAs and targets in ginger and mango ginger leaf and rhizome tissues at different time intervals post inoculation with *R. solanacearum.***A** 4CLL1 & ath-miR1886, **B** ABCC5 & mtr-miR5261, **C** PRT6 & osa-miR169, **D** RPS2 & gra-miR482, **E** FRS6 & ppt-miR1223, **F** AAP4 & mtr-miR164, **G** WRKY19 & aly-miR398, **H** ABCG11 & gma-miR4415 and **I** DNAJ1 & ath-miR167. Values are the means ± standard deviation of three independent experiments involving three replicates. Values superscripted with the different letters are significantly different based to Tukey’s HSD at *P* ≤ 0.05

The qPCR analysis validated nine selected miRNA target genes (4CLL1, ABCC5, PRT6, RPS2, FRS6, AAP4, WRKY19, ABCG11 and DNAJ1) and demonstrated their differential expression in leaf and rhizome tissues of both ginger and mango ginger during bacterial wilt infection. Among the nine miRTGs, seven were upregulated in leaves and eight in rhizome tissues of ginger. In mango ginger miRTGs, six in leaves and seven in rhizome were upregulated. Among the miRTGs, four (4CLL1, ABCC5, PRT6 and RPS2) showed upregulation in both the tissues of ginger and mango ginger. The miRTGs, FRS6 and WRKY19, were upregulated in ginger but downregulated in mango ginger, whereas AAP4 showed downregulation in ginger and upregulation in mango ginger. The ABCG11 was upregulated in all three samples except ginger leaves. In DNAJ1, an upregulation was observed in all samples except mango ginger leaves (Table 5 and Fig. 7).

**Table 5:** List of upregulated and downregulated genes

	Upregulated	Downregulated
Ginger leaves	4CCL, ABCC5, PRT6, RPS2, FRS6, WRKY19, DNAJ1	AAP4, ABCG11
Mango ginger leaves	4CCL, ABCC5, PRT6, RPS2, AAP4, ABCG11	FRS6, WRKY19, DNAJ1
Ginger rhizome	4CCL, ABCC5, PRT6, RPS2, FRS6, WRKY19, DNAJ1, ABCG11	AAP4
Mango ginger rhizome	4CCL, ABCC5, PRT6, RPS2, DNAJ1, ABCG11, AAP4	FRS6, WRKY19

Stem-loop PCR was performed to analyse the expression of nine miRNAs (ath-miR1886, mtr-miR5261, osa-miR169, gra-miR482, ppt-miR1223, mtr-miR164, aly-miR398, gma-miR4415 and ath-miR167). Among them, mtr-miR164 and ppt-miR1223 showed upregulation in both the tissues of ginger and mango ginger. However, ppt-miR1223 downregulated in ginger rhizome after 8 h post-inoculation. The miRNAs, gra-miR482 and ath-miR167 upregulated in both tissues of mango ginger but downregulated in ginger in all the time intervals. Ath-miR1886 and aly-miR398 were upregulated only in mango ginger rhizome post inoculation (Fig. 7).

The correlation analysis was carried out using the Pearson correlation coefficient to understand the influence of miRNAs and their targets. The results of miRNAs showing Pearson correlation coefficient with its corresponding miRNA target of more than 0.9 is given in Table 6. However, the expression profiles of all the miRNAs were not perfectly negative to its corresponding targets.

**Table 6:** Correlation analysis of miRNAs expression profiles and their target genes in response to *Ralstonia solanacearum*

Target gene	Protein	Selected miRNA	Pearson correlation coefficient			
			Ginger leaves	Ginger rhizome	Mango ginger leaves	Mango ginger rhizome
RPS2	NBS-LRR	miR482	-0.94	-0.92	-0.92	-0.90
FRS6	FAR1-related sequence 6	miR-1223	-0.92	-0.91	0.99	-0.91
ABCC5	ABC transporter	miR-5261	-0.94	-0.91	-0.97	-0.95
PRT6	Ubiquitin protein ligase	miR-169	-0.95	-0.98	-0.95	-0.98
ABCG11	ABC transporter	miR-4415	-0.97	-0.97	-0.97	-0.94
AAP4	Amino acid-proton symporter	miR-164	0.90	-0.91	-0.97	-0.93
DNAJ1	Heat shock protein	miR-167	-0.93	-0.90	-0.95	-0.97
WRKY19	Transcription factor	miR-398	-0.912	-0.94	-0.97	-0.90
4CLL1	4-coumarate-CoA ligase	miR-1886	-0.96	-0.98	-0.91	-0.97

## Discussion

### Strategies to reveal the disease-resistant mechanism of mango ginger

Ginger, a herbaceous tropical perennial crop, belongs to the family *Zingiberaceae* [20]. Among the diseases that affect ginger, bacterial wilt caused by *R. solanacerum* is one of the major production constraints in several regions of the world [21]. Even though there are several released varieties in ginger, none of them is resistant to *R. solanacearum* infection due to lack of genetic variability. Among the primary and secondary gene pool, the mango ginger (*C. amada*) exhibited immune response to *R. solanacearum* [22]. Initially, PR5 genes in ginger and mango ginger were cloned, and their expression was analyzed to understand their role in disease resistance [23]. Later, number of candidate genes were identified based on transcriptome-wide sequencing of ginger and mango ginger under bacterial wilt [10]. Moreover, a web source was generated, which provides public access to the ginger and mango ginger transcriptome database. Here, we employed the same RNA-Seq data to identify the miRNAs and their targets in two ginger species upon *R. solanacearum* infection. Also, to determine the differential expression and regulation of miRNAs and their targets. The study is expected to deliver several insights in understanding the miRNA regulatory network in ginger and mango ginger against *R. solanacearum*.

### Differential expression and functional annotation of DEmiRTGs

A total of 4736 and 4485 DEmiRTGs were identified in ginger and mango ginger in response to *R. solanacearum* (Fig. 1). Functional annotation results showed that the term “defence response’’ had higher enrichment in mango ginger than ginger. Among the upregulated common miRTGs and miRTGs exclusively expressed in mango ginger, maximum enrichment was observed in the “defence response”. Moreover, there were 215 miRTGs which was upregulated in mango ginger but downregulated in ginger. Among them 23 GO terms were related to “defence response”. Also “defence response towards bacterium” was the major GO term in all cases. The GO analysis clearly revealed the activation and higher expression of several defence-related proteins, especially defence response proteins towards bacterium in resistant ginger. KEGG pathway analysis showed that carbohydrate metabolism terms in mango ginger were higher when compared to ginger. It was previously reported that carbohydrate metabolism was disturbed during *R. solanacearum* infection in tomato [24]. A well-established activation of carbohydrate metabolism pathway genes may be one reason for bacterial wilt resistance in mango ginger.

### Validation of differentially expressed selected miRTGs

This study mainly focused on the identification miRTGs, and their potential miRNAs involved in regulating bacterial wilt defence reactions. We have selected nine miRTGs and validated their expression. These selected miRTGs were previously reported to have a significant role in plant-pathogen interaction during biotic stress.

4CLL (4-coumarate-CoA ligase) is considered an essential component in the phenylpropanoid pathway [25]. It primarily participates in the biosynthesis of p-coumaroyl-CoA, a precursor biosynthesis of several plant metabolites, including coumarin [26]. Coumarin accumulation is considered one of the most critical responses in infection with various viruses, fungi, and pathogens [27, 28]. The role of coumarin in defence response against *R. pseudosolanacearum* infection in tobacco has been reported previously [29]. In our study, the expression of 4-coumarate-CoA ligase (4CLL1) was upregulated in ginger and mango ginger during bacterial wilt infection. Moreover, the expression levels were higher in mango ginger compared to ginger in both tissues. This clearly shows the role of this gene during bacterial wilt (Fig. 7A).

Though initially identified as transporters that play in detoxification processes, later ABC transporters were shown to be required in stress responses, pathogen interaction [30]. In the present study, we compared the expression of two ABC transporter genes, ABCC5 and ABCC11, during bacterial wilt among ginger species. ABCC5 encodes a high-affinity inositol Hexa kis phosphate transporter, which plays a part in guard cell signalling and phytate storage [31]. This protein was reported to play a role in the defence mechanism in several plants [31, 32]. ABCC11 has reported being involved in developmental plasticity and stress responses [33]. ABCC5 and ABCC11 were differentially expressed among resistant and susceptible gingers. However, their expression was more evident in rhizome than leaves in both ginger species. Since bacterial wilt is a rhizome borne disease in ginger, these genes may play a critical role in disease resistance (Fig. 7B & H). Similarly, a difference in expression of ABC transporter genes was shown in *Solanum* sps when *Globodera pallida* infected resistant and susceptible plant roots [34].

PRT6 is a ubiquitin-protein ligase that is a component of the N-end rule pathway. It regulates the biosynthesis of plant-defence metabolites such as glucosinolates, and phytohormone jasmonate which play a crucial role in plant immunity [35]. In our experiment, PRT6 had higher expression in mango ginger leaves and rhizome tissues when compared to ginger (Fig. 7C). PRT6 mutants in Arabidopsis also showed higher susceptibility than wild type when infected with *R. solanacearum* [35].

NBS-LRR genes are one of the most studied disease resistance gene family in plants [36]. Several studies have been carried out in these genes in giving resistance in wheat, cassava, sugarcane, rice, apple, coconut [37, 38]. They are involved in detecting various biotic stresses such as pathogens, including bacteria, viruses, fungi, nematodes, insects, and oomycetes. Their action can lead to plant cell death in the typical hypersensitive response [39]. We compared the expression of an NBS-LRR protein, RPS2, which was also well characterized in other plants [40, 41]. There was a significant difference in the expression of RPS2 in mango ginger when compared to ginger (Fig. 7D). Moreover, the expression was higher in rhizome when compared to leaves. It was reported earlier that overexpression of RPS2 activates a downstream defence response pathway in Arabidopsis [42].

FRS6 (FAR1-related sequence 6) is a protein essential for phytochrome A controlled far-red responses in Arabidopsis. Loss-of-function mutants display elongated hypocotyls, specifically under continuous FR light (FRc) [43]. It was reported that during heat stress, this gene was downregulated in conic seagrass, *Posidonia oceanica* [44]. In agreement with previous reports, the FRS6 was downregulated in resistant ginger and upregulated in susceptible ginger (Fig. 7E).

AAP4 is an amino acid permease which is an amino acid-proton symporter. It is a stereospecific transporter and has broad specificity for neutral amino acids, such as alanine, asparagine, and glutamine. It was reported that amino acid transporters play a crucial role in plant defence by controlling amino acid transport [45, 46]. Significantly differential expression of AAP4 among tissues and ginger species was observed in our experiment (Fig. 7F). Thus, amino acid transporter might have a significant role in defence response against *R. solanacearum.*

WRKY19 is a transcription factor that acts as a disease resistance protein with a serine/threonine-protein kinase activity [47]. The TIR-NB-LRR pair DSC1 and WRKY19 contributed to basal immunity in Arabidopsis against the root-knot nematode *Meloidogyne incognita* [48]. Also, overexpression of wheat WRKY gene TaWRKY19 increased the salt, drought, and freezing tolerance in transgenic plants [49]. Our study showed high expression in ginger leaves when compared to mango ginger (Fig. 7G). Nevertheless, in the case of the rhizome, higher expression of WRKY 19 was observed in mango ginger. Thus, WRKY19 might have some critical role in defence response.

DNAJ1 are heat shock proteins (HSPs) that are molecular chaperones known for controlling inappropriate aggregation, folding, misfolding, and unfolding several proteins. They are reported to be involved in hyperosmotic and heat shock by preventing the aggregation of stress-denatured proteins and disaggregating proteins [50]. In this study, DNAJ1 was differentially expressed among different tissues of ginger species (Fig. 7I). These results proved its role in defence is tissue-specific and different in ginger species.

### Comparison and validation of expression of selected miRNAs

Several reports of plant miRNA involvement in regulating signalling pathways, NBS-LRR gene expression, and ROS pathways during pathogen infestation are available. Here we studied the role of nine miRNAs during bacterial wilt in susceptible and resistant ginger species. As mentioned earlier, even though each miRTGs had targets for several miRNAs, data of miRNAs showing Pearson correlation coefficient with its corresponding miRNA target of more than 0.9 were only analyzed.

miR1886 was found to play an important role in abiotic and biotic stress in various plants. miR1886 was less expressed in response to ABA treatment related to an increase in Cf4CL transcripts [51]. In this study, ath-miR1886 targeted 4CLL1 in ginger and mango ginger. Ath-miR1886 was upregulated in mango ginger rhizome and downregulated in ginger leaves and rhizomes. This suggests that it is tissue-specific and species-specific. However, the expression difference among ginger species was a strong indication of miR1886’s role in bacterial wilt resistance (Fig. 7A).

Several extended studies were carried out to understand the role of miRNA mediated regulation of ABC transporters. Here we studied mtr-miR5261 and gma-miR4415, which targets ABCC5 and ABCG11, respectively. Both miRNAs were previously reported to play defence response during biotic and abiotic stress [52–54]. Even though both miRNAs were expressed differentially among ginger species, gma-miR4415 seems to play effectively in defence against bacterial wilt since its expression was 10 to 20 times higher in rhizome than leaves of resistant one (Fig. 7B&H).

Osa-miR169, which targets PRT6, is a conserved miRNA family reported in plant growth, development and responses induced by stress. In rice, osa-miR169 negatively regulate the immune response against blast fungus, *Magnaporthe oryzae*. It proved that regulation was made by repressing the expression of nuclear transcription factor subunit alpha (NF-YA) genes. Moreover, osa-miR169 overexpressed transgenic rice lines were hyper susceptible to *M. oryzae* infection [55]. Our study clearly showed that osa-miR169 was down-regulated in both ginger and mango ginger, but less expression in ginger than mango ginger (Fig. 7C). Also, downregulation of this miRNA in the early stages proves its crucial role during *R. solanacerum* infection.

Gra-miR482 is an ancient and extensive family of miRNA present in all land plants. In cotton, it regulated the target site by cleaving and inhibiting the expression of target nucleotide-binding sites and leucine-rich repeat (NBS-LRR) [56]. In this study, the gra-miR482 mainly targets RPS2. Gra-miR482 was down-regulated in leaves and rhizomes of ginger but upregulated in mango ginger, even in the initial hours of the infection (Fig. 7D). These results imply that miR482 regulates the defence response of the NBS-LRR gene in ginger species.

Ppt-miR1223 targets mainly genes related to nucleic acid binding and secondary metabolic process. In ginger it targets mainly FRS6. miR1223 was reported previously in *Allium cepa* [57] and *Physcomitrella patens* [58]. This miRNA upregulated in the rhizome of both ginger and mango ginger, till 12 h post-infection (Fig. 7E). However, later this miRNA was down regulated in ginger rhizomes, indicating its role during the early phase of infection. Several miRNAs showed a differential expression in the early and late phase of the disease [59].

Mtr-miR164 was conserved, highly expressed in fruits, and validated to target a subset of the NAC-domain transcription factor gene family such as AAP4 [60, 61]. Aly-miR398 was a conserved miRNA identified in Arabidopsis which targets WRKY19 [62]. Both were expressed tissue specifically and more in the rhizomes of resistant ginger compared to susceptible one (Fig. 7F &G). However, the role played by these miRNAs on disease resistance or susceptibility is not clearly understood.

The ath-miR167 was reported to be important in correcting gene expression patterns and fertility in both ovules and anthers in Arabidopsis [63]. In this study it cleaved DNAJ1. Among the ginger species, the higher expression in mango ginger revealed its vital role in resistance (Fig. 7I).

## Conclusions

Our study utilized the already available RNA-Seq data to identify and compare DEmiRTGs and their miRNAs in ginger and mango ginger against *R. solanacearum*. This strategy aided in developing a new miRNA seq data of 4736 and 4485 DEmiRTGs in ginger and mango ginger, respectively. Functional annotation results showed that several defence response terms were enriched specifically in resistant ginger. KEGG analysis revealed the crucial role of carbohydrate metabolism during bacterial wilt infection in ginger. Additionally, we identified nine miRNA/miRTGs key candidate pairs in response to *R. solanacearum* infection in ginger. Our results paved the way in understanding significant miRNA-mediated posttranscriptional regulation of bacterial wilt resistance in mango ginger. The data set generated is further helpful in exploring miRNAs function in improving ginger resistance to bacterial wilt.

## Methods

### miRNA target and miRNA prediction

To evaluate miRNAs’ role in response to bacterial wilt infection in ginger (susceptible) and mango ginger (resistant), we have used already available RNA-Seq data of ginger (PRJNA311170), and mango ginger (PRJNA315599) challenged with Ralstonia. The leaf tissues of three biological replicates were sampled over a 72 h period post-inoculation and pooled before RNA preparation. The two RNA libraries were constructed and sequenced using Illumina RNA-Seq method [64]. After sequencing, high-quality reads were retained after removing the adapter, low-quality sequences and PCR duplicates from the raw data using FASTQ Quality Check [65].

Those clean reads were utilized here to reconstruct the transcriptome de novo. Trinity-v2.11.0 software with the parameters: minimum contig length of 100 bases and average fragment length 300 bp was used [66]. These two predicted transcriptome data were utilized to predict conserved miRTGs and identify the miRNAs for each target. Both were carried out using the psRNATarget server [67]. In this research, the following default parameters were used for identifying potential miRTGs: (1) maximum expectation 3; (2) length for complementarity scoring (hsp size) 20; (3) target accessibility—allowed maximum energy to unpair the target site (UPE) 25; (4) flanking length around target site for target accessibility analysis 17 bp upstream and 13 bp downstream and (5) range of central mismatch leading to translational inhibition 9–11 nucleotides. For the prediction of miRNAs for each miRTGs, above mentioned psRNATarget server was used with default parameters.

### Differential expression and functional analysis

Differential expression was conducted using edgeR [68] with significant miRTGs based on FDR-corrected *p*-value <  = 0.05. The number of reads mapped and Reads Per Kilobase of the transcript, per Million mapped reads (RPKM) were used to calculate fold change (FC). Transcripts with FC >  = 0.8 and *P* value <  = 0.05 was considered as upregulated and FC <  = -0.8 and *P* value <  = 0.05 were considered as downregulated. Blast2GO software with default settings was used in Gene Ontology (GO) annotation and KEGG (Kyoto Encyclopedia of Genes and Genomes) pathway analysis to identify the putative biological functions and pathways of the DEmiRTGs. Initially, BLASTX aligned (E ≤ 1e-6) contigs to the NCBI non-redundant protein database and later GO terms were retrieved for each BLAST hit with default parameters. KEGG maps were also retrieved for each query sequence as per instructions [69].

### Construction of regulatory network using miRNA-mRNA

Visualization of the regulatory network between miRNA and mRNA was constructed using Cytoscape 3.7.1 [70].

### Plant material

For experimental validation, ginger (susceptible) and mango ginger (resistant) varieties used were IISR Varada and Amba, respectively. Disease-free rhizomes were obtained from the Experimental Farm of ICAR-Indian Institute of Spices Research, Peruvannamuzhi, Kerala, India. Sterile distilled water was used to wash the rhizome thoroughly. They were then planted in autoclaved perlite. The plants were maintained in the net house of the ICAR-Indian Institute of Spices Research, Kozhikode, Kerala.

### Inoculum preparation and inoculation procedure

Initially, fresh cultures *R. solanacearum* strain GRs Mnt2 (virulent) grown using Casamino acid-Peptone-Glucose (CPG) medium were inoculated in CPG broth kept at 28 °C for 16 h with continuous shaking. Forty-five-day old, same sized plants were carefully removed from perlite and washed. Plants were incubated for five days in a beaker containing 150 ml virulent broth under greenhouse conditions. Similarly, fresh plants were maintained in sterile water as control. Plant tissues (leaves and rhizome) were collected form different time intervals post-inoculation (1, 4, 8, 12, 24, 48, 72, 96, and 120-h post-inoculation (hpi)).

### Expression validation of using qPCR

Here quantitative RT-PCR (qRT-PCR) was performed to validate the expression of miRTGs and miRNAs on the Rotor-Gene Q real-time PCR system (Qiagen, USA). For each sample, there were three biological replicates. Stem-loop qRT-PCR [71] for miRNA and regular qPCR [72] for target genes were conducted as described previously. The fold change of the transcripts was calculated relative to the control (0 hpi) using the 2^− ddCt^ method using β-actin as an internal control. List of primers used are available in Tables 2 and 3.

### Statistical analysis

To evaluate the statistical validity of results, all experiments were performed three times, each having five replicates. IBM SPSS Statistics 24 was used for statistical analysis. Data shown are the mean value ± standard deviation of three independent experiments. One way ANOVA along with Tukey posthoc test at *P* < 0.05 level of significance was carried out. Correlation analysis between selected miRNAs and their targets were carried out using the Pearson coefficient.

## Supplementary Information

**Additional file 1.** List of miRTGs with their corresponding miRNAs.

## Data Availability

The RNA-seq data used in this study was available through the NCBI under accession number PRJNA315599 (mango ginger) and PRJNA311170 (ginger). We have a permission to collect the plant samples we used in this study. All the data and materials that are required to reproduce these findings can be shared by contacting the corresponding author, Duraisamy Prasath (Prasath.D@icar.gov.in).

## Abbreviations

miRNA: MicroRNA
DEmiRTG: Differentially expressed miRNA target gene
miRTGs: MiRNA target genes
Zo: *Zingiber officinale*
Ca: *Curcuma amada*
*R. solanacearum*: *Ralstonia solanacearum*
